# SARS-CoV-2 Vaccine Safety and Autoimmune Response

**DOI:** 10.3390/vaccines12030334

**Published:** 2024-03-20

**Authors:** Yu-Chang Tyan, Shih-Chang Chuang, Tzu-Chuan Ho, Kuo-Pin Chuang, Ming-Hui Yang

**Affiliations:** 1 Department of Medical Imaging and Radiological Sciences, Kaohsiung Medical University, Kaohsiung 807, Taiwan; 2 Center for Cancer Research, Kaohsiung Medical University, Kaohsiung 807, Taiwan; 3 Graduate Institute of Animal Vaccine Technology, College of Veterinary Medicine, National Pingtung University of Science and Technology, Pingtung 912, Taiwan; 4 Department of Medical Research, Kaohsiung Medical University Hospital, Kaohsiung 807, Taiwan; 5 Center for Tropical Medicine and Infectious Disease Research, Kaohsiung Medical University, Kaohsiung 807, Taiwan; 6 Division of General & Digestive Surgery, Department of Surgery, Kaohsiung Medical University Hospital, Kaohsiung Medical University, Kaohsiung 807, Taiwan

Coronavirus disease 2019 (COVID-19) is a global public health crisis. Its antiviral and immunomodulatory treatments are adapted for symptomatic relief, but it is vaccination that is needed to significantly impact the disease’s spread. However, after the extensive usage of SARS-CoV-2 vaccination, various adverse effects and complications have been described. This Special Issue, “Safety and Autoimmune Response to SARS-CoV-2 Vaccination”, provides a comprehensive compilation of research articles that delve into various aspects of vaccine safety and autoimmune response, offering an in-depth understanding of such critical fields. The collection of papers sheds light on the latest trends, challenges, and advancements in the domain.

Notably, several articles evaluate vaccine safety, leveraging large-scale vaccination data to discern potential correlations between vaccine administration and adverse events. Through rigorous analysis, these evidence-based approaches may help to inform the public that the benefits of immunization far outweigh the potential risks. Furthermore, research is included that focuses on the safety of vaccine administration within specific populations, including immunocompromised individuals and pregnant women, highlighting tailored strategies that optimize safety while maximizing the protective effects of vaccines.

Vaccine safety has been studied. The four COVID-19 vaccines (AstraZeneca, Pfizer, Moderna, and Janssen) have shown some common local and systemic reactions, such as fever, chills, fatigue, headache, and muscle pain, that have frequently been seen after vaccinations. Data have suggested that the vaccines’ efficacy against COVID-19 infection is above 60% and have also aided evidence-based decision-making [1]. It has been reported that antibodies against platelet factor 4 (anti-PF4) are pathogenic and are induced after receiving vaccinations [2]. However, Zhang and coworkers reported that pathogenic platelet-activating antibodies were not frequently found after the administration of vaccinations [3]. In fact, the frequency of positively detecting anti-PF4 antibodies after receiving the first dose of the ChAdOx1 nCoV-19 vaccination was low, and the second dose of the vaccination also did not increase the antibody level. It was relatively safe for individuals to receive a booster dose, even asymptomatic people with previously positive anti-PF4 antibodies [3]. Thus, positive PF4 results should be interpreted with caution in clinically asymptomatic individuals who have recently been vaccinated against SARS-CoV-2 [4]. The effects of heterologous immunization against SARS-CoV-2 were also investigated. Compared with participants having homologous vaccination, serious adverse events in individuals with ChAdOx1/BNT162b2, ChAdOx1-S/mRNA-1273, or BNT162b2/ChAdOx1-S were not reported. In addition, participants with the above heterologous regimen, compared with those with two doses of ChAdOx1-S, showed stronger immune responses against SARS-CoV-2, including higher levels of responsive antibodies or elevation of the spike-specific T-cell quantity [5]. The safety profiles of authorized vaccines were inspected. Pain at the injection site, fever, headache, fatigue, and muscle and joint pain were the common side effects. Intriguingly, females perceived more side effects compared with males, and subjects under sixty were more likely to experience side effects than the participants in other age groups [6]. When monitoring adverse events following immunization (AEFI) with four COVID-19 vaccines (AstraZeneca, Pfizer, Moderna, and Novavax) across a four-dose schedule (primary doses 1, 2, and 3, and booster), the Moderna vaccinations were associated with higher proportions of AEFI compared with the others. Interestingly, Novavax was reported to have a higher proportion of AEFI when newly introduced. Thus, a nocebo effect may be observed if short-term surveillance is utilized [7]. COVID-19 vaccine modalities had poor effectiveness for subjects with hematological conditions since more than half of the subjects only produced a limited antibody response to the vaccine. However, for patients with myelodysplastic syndrome (MDS), specific vaccine treatments may be selected since the patients’ stronger immune responses have been found to be brand-dependent [8]. Hence, the benefit of vaccination can still outweigh the risk of side effects.

Nonetheless, concerns of autoimmune responses following SARS-CoV-2 vaccination have been raised. Autoimmune encephalitis is one of the reported neurological adverse effects following COVID-19 vaccination, and its treatment is different from autoimmune dementia. The possibility of rare autoimmune reactions following this novel vaccination should be noted [9]. Compared with other systemic autoimmune and inflammatory disorders and healthy individuals, patients with systemic sclerosis do not have higher risks of delayed post-COVID-19 vaccine-related delayed adverse events. However, diffuse cutaneous phenotypes and those concomitant with autoimmune diseases (e.g., myositis and thyroid disease) may raise the risk of delayed adverse events to a slight degree [10]. A concern is that people with multiple sclerosis may have a relapse post-COVID-19 vaccination. Nabizadeh and coworkers found that most of the patients they studied did not experience post-vaccination relapses. Moreover, disease progression and medication before or during the vaccination could play a more vital role in relapse incidence than vaccination itself [11]. Incidences of autoantibody seroconversion after COVID-19 vaccination are low and their number may become negligible. In addition, no new-onset autoimmune inflammatory rheumatic diseases have been detected [12]. COVID-19 vaccination is relatively safe for autoimmune inflammatory rheumatic disease patients. For zoster virus reactivation, compared with healthy controls, autoimmune inflammatory rheumatic diseases by themselves do not increase the risk of reactivation following COVID-19 vaccination [13]. Nevertheless, being concomitant with diabetes mellitus, chronic hepatitis B virus infection, and the usage of immunosuppressants (mycophenolate mofetil) were independent risk factors for varicella zoster virus reactivation [13]. The reactivation of herpes zoster following COVID-19 vaccination has occurred in a very small percentage of patients with autoimmune inflammatory rheumatic diseases (AIIRDs) [14]. However, the relationship between COVID-19 vaccination and the reactivation of zoster needs to be clarified. In addition, severe adverse events for cardiovascular, renal, and autoimmune diseases and reproductive health issues have been reported regardless of the causality. Accordingly, individuals with pre-existing conditions need to be cautious of COVID-19 vaccination [15]. The development of autoimmune skin diseases after COVID-19 vaccination, such as the development, worsening, or recurrence of alopecia areata, has been reported. When properly treated, three-fourths of these patients showed hair regrowth [16]. Environmental exposure, such as from novel vaccines, may trigger autoimmune endocrine diseases (AIEDs), including Graves’ disease (GD). It has also been found that an individual’s predisposition may be influential for the development of AIEDs [17]. Although post-vaccine optic neuritis occurring in patients with autoimmune disease is rare, individuals who are predisposed to immune-mediated disorders need to be carefully assessed before COVID-19 vaccination. However, the mechanism for this is unclear and the linkage is not proved [18]. As an example of a study on vaccination-induced autoimmunity, Guo and co-workers outlined possible mechanisms, such as molecular mimicry/immune cross-reaction, activation by bystanders, and the effects of adjuvants [19]. COVID-19 vaccine-associated lymphadenopathy is influenced by age and gender, and reactive changes within lymph nodes significantly contribute to its development. Notably, shorter duration post-vaccination and robust B cell germinal center response are linked to reduced C19-VAL incidence [20]. Table 1 shows the possible pathomechanisms of adverse effects.

Interestingly, recent studies have revealed the additional benefits of vaccination. A study, involving more than one million COVID-19 and three million non-COVID-19 subjects, showed that COVID-19 vaccination decreased the risk of pemphigoid, Graves’ disease, anti-phospholipid antibody syndrome, immune-mediated thrombocytopenia, systemic lupus erythematosus, and other autoimmune arthritis types [21]. In addition, Chen and coworkers reported that COVID-19 vaccines induced a hepatoprotective effect in non-alcoholic fatty liver disease patients during SARS-CoV-2 infection, in which vaccines might have played a role in maintaining hepatic homeostasis during SARS-CoV-2 infection [22]. It was found that for the vaccinated non-alcoholic fatty liver disease patients, liver function abnormality was less frequent, the incidence of abnormal bilirubin levels was lowered, and the viral shedding duration was shorter during the infection.

In summary, SARS-CoV-2 vaccines have played a pivotal role in controlling the COVID-19 pandemic. While they are generally safe and effective, concerns about vaccine-induced autoimmune responses persist. An extensive overview of vaccine safety profiles, vaccine-related autoimmune responses, adverse effects, underlying molecular mechanisms, and strategies for mitigating risks have been discussed. These findings contribute essential knowledge for guiding vaccination policies, enhancing monitoring strategies, and addressing potential challenges. In addition, recent studies have revealed the additional benefits of vaccination. The insights gained are invaluable for healthcare professionals and researchers, ensuring a well-informed and proactive approach to vaccination safety in the ongoing fight against the COVID-19 pandemic.

## Figures and Tables

**Table 1:** Adverse effects following COVID-19 vaccination.

Adverse Effects	Major Finding	Pathomechanisms	Reference
Local and systematic reactions	Higher frequency of systematic reactions in heterologous vaccination.	Unknown	[5]
	Higher frequency of local reactions after the 1st dose of vaccination. Higher frequency of systematic reactions after the 3rd dose of mRNA vaccine.	Cytokines induced by the presence of already-synthesized immune cells	https://doi.org/10.3390/vaccines11020281
	High percentage of local and systematic reactions was found in Moderna vaccination after 2nd and 3rd doses and booster.	Unknown	https://doi.org/10.3390/vaccines10122017
	Moderna and AstraZeneca cause local and systematic reactions more frequently than the Pfizer-BioNTech vaccine.	Unknown	https://doi.org/10.3390/vaccines11020231
Lymphadenopathy	High relation between reactive change and lymphadenopathy development. Incidence is negative between day post-vaccination and B cell germinal center response.	Suspected strong vaccine immune response	[20]
Graves’ disease	New diagnoses and recurrences of Grave’s disease are raised after vaccination.	Adjuvant-induced autoimmune or inflammatory syndrome. Individual predisposition.	https://doi.org/10.3390/vaccines10091445
Alopecia areata	Description of the occurrence of alopecia areata following vaccination.	Unknown	https://doi.org/10.3390/vaccines10091467
Non-communicable disease	Vaccination-related disease: cardiovascular disease, acute kidney disease, neurological disorders, psychiatric and mental disorders.	Unknown	https://doi.org/10.3390/vaccines11020208
Autoimmune encephalitis	Cognitive deficits frequently occurred in autoimmune encephalitis after vaccination.	Unknown	[9]

## References

[1] Hernández A.F., Calina D., Poulas K., Docea A.O., Tsatsakis A.M. (2021). Safety of COVID-19 vaccines administered in the EU: Should we be concerned?. Toxicol. Rep..

[2] Scully M., Singh D., Lown R., Poles A., Solomon T., Levi M., Goldblatt D., Kotoucek P., Thomas W., Lester W. (2021). Pathologic Antibodies to Platelet Factor 4 after ChAdOx1 nCoV-19 Vaccination. N. Engl. J. Med..

[3] Zhang Y., Bissola A.L., Treverton J., Hack M., Lychacz M., Kwok S., Arnold A., Nazy I. (2024). Vaccine-Induced Immune Thrombotic Thrombocytopenia: Clinicopathologic Features and New Perspectives on Anti-PF4 Antibody-Mediated Disorders. J. Clin. Med..

[4] Thiele T., Ulm L., Holtfreter S., Schönborn L., Kuhn S.O., Scheer C., Warkentin T.E., Bröker B.M., Becker K., Aurich K. (2021). Frequency of positive anti-PF4/polyanion antibody tests after COVID-19 vaccination with ChAdOx1 nCoV-19 and BNT162b2. Blood.

[5] Ho T.-C., Chen Y.-M.A., Chan H.-P., Chang C.-C., Chuang K.-P., Lee C.-H., Yuan C.-H., Tyan Y.-C., Yang M.-H. (2021). The Effects of Heterologous Immunization with Prime-Boost COVID-19 Vaccination against SARS-CoV-2. Vaccines.

[6] Haider S.M.S., Alvi S.A., Khan H., Majeed R., Syed T., Anwar A., Hashmi A.A. (2023). Common Side Effects of Pfizer COVID-19 Vaccine: An Experience From Pakistan. Cureus.

[7] Chi W.Y., Li Y.D., Huang H.C., Chan T.E.H., Chow S.Y., Su J.H., Ferrall L., Hung C.F., Wu T.C. (2022). COVID-19 vaccine update: Vaccine effectiveness, SARS-CoV-2 variants, boosters, adverse effects, and immune correlates of protection. J. Biomed. Sci..

[8] Beltrami-Moreira M., Bussel J.B. (2022). A narrative review of anti-SARS-CoV-2 vaccines and immune thrombocytopenia: Be aware, but reassured. Clin. Adv. Hematol. Oncol..

[9] Huang Y.-F., Ho T.-C., Chang C.-C., Shen D.H.-Y., Chan H.-P., Chuang K.-P., Tyan Y.-C., Yang M.-H. (2022). A Rare Adverse Effect of the COVID-19 Vaccine on Autoimmune Encephalitis. Vaccines.

[10] Panchawagh S., Bohdana D., Kuwana M., Yoshida A., Yomono K., Pauling J.D., Makol A., Kadam E., Day J., Chatterjee T. (2023). Delayed adverse events following COVID-19 vaccination in patients with systemic sclerosis and other autoimmune diseases: A substudy of the COVAD-2 cohort. Rheumatol. Int..

[11] Nabizadeh F., Ramezannezhad E., Kazemzadeh K., Khalili E., Ghaffary E.M., Mirmosayyeb O. (2022). Multiple sclerosis relapse after COVID-19 vaccination: A case report-based systematic review. J. Clin. Neurosci..

[12] Furer V., Eviatar T., Zisman D., Peleg H., Paran D., Levartovsky D., Zisapel M., Elalouf O., Kaufman I., Meidan R. (2021). Immunogenicity and safety of the BNT162b2 mRNA COVID-19 vaccine in adult patients with autoimmune inflammatory rheumatic diseases and in the general population: A multicentre study. Ann. Rheum. Dis..

[13] Chen J., Li F., Tian J., Xie X., Tang Q., Chen Y., Ge Y. (2023). Varicella zoster virus reactivation following COVID-19 vaccination in patients with autoimmune inflammatory rheumatic diseases: A cross-sectional Chinese study of 318 cases. J. Med. Virol..

[14] Furer V., Zisman D., Kibari A., Rimar D., Paran Y., Elkayam O. (2021). Herpes zoster following BNT162b2 mRNA COVID-19 vaccination in patients with autoimmune inflammatory rheumatic diseases: A case series. Rheumatology.

[15] Noushad M., Nassani M.Z., Samran A., Dimashkieh M.R., Al-Awar M.S. (2023). COVID-19 and herpes zoster: A call to action. Front. Public Health.

[16] Rossi A., Magri F., Michelini S., Caro G., Di Fraia M., Fortuna M.C., Pellacani G., Carlesimo M. (2021). Recurrence of alopecia areata after covid-19 vaccination: A report of three cases in Italy. J. Cosmet. Dermatol..

[17] Triantafyllidis K.K., Giannos P., Stathi D., Kechagias K.S. (2022). Graves’ disease following vaccination against SARS-CoV-2: A systematic review of the reported cases. Front. Endocrinol..

[18] Pirani V., Pelliccioni P., Carpenè M.J., Nicolai M., Barbotti F., Franceschi A., Mariotti C. (2023). Optic neuritis following COVID-19 vaccination: Do autoimmune diseases play a role?. Eur. J. Ophthalmol..

[19] Guo M., Liu X., Chen X., Li Q. (2023). Insights into new-onset autoimmune diseases after COVID-19 vaccination. Autoimmun. Rev..

[20] Ho T.-C., Shen H.-Y., Chang C.-C., Chan H.-P., Chuang K.-P., Yuan C.-H., Chen C.-N., Yang M.-H. (2023). Immune Response Related to Lymphadenopathy Post COVID-19 Vaccination. Vaccines.

[21] Peng K., Li X., Yang D., Chan S.C.W., Zhou J., Wan E.Y.F., Chui C.S.L., Lai F.T.T., Wong C.K.H., Chan E.W.Y. (2023). Risk of autoimmune diseases following COVID-19 and the potential protective effect from vaccination: A population-based cohort study. eClinicalMedicine.

[22] Chen Z., Tang W., Feng N., Lv M., Meng F., Wu H., Zhao Y., Xu H., Dai Y., Xue J. (2024). Inactivated vaccines reduce the risk of liver function abnormality in NAFLD patients with COVID-19: A multi-center retrospective study. eBioMedicine.

